# Supplementary material for: Inflammasome Activity in the Skeletal Muscle and Heart of Rodent Models for Duchenne Muscular Dystrophy
Source: Int J Mol Sci. 2023 May 9;24(10):8497. doi: 10.3390/ijms24108497 (PMC10218525; doi:10.3390/ijms24108497)
Supplement: Supplementary file 1 [file ijms-24-08497-s001.zip › ijms-2334704-supplementary.pdf]

Supplementary Materials

Inflammasome activity in the skeletal muscle and heart of rodent models for Duchenne muscular dystrophy

Short title: Inflammasomes in Duchenne muscular dystrophy

Authors: Zsófia Onódi [1,2,3]*, Petra Lujza Szabó [4,*], Dániel Kucsera [1,2,3], Tamás Gábor Szabó [4], Péter Pokreisz [4], Christopher Dostal [4], Karlheinz Hilber [5], Gavin Y Oudit [6], Bruno K. Podesser [4], Péter Ferdinandy [1,7], Zoltán V. Varga [1,2,3,#,†], Attila Kiss [4,#]

1 Department of Pharmacology and Pharmacotherapy, Semmelweis University, Budapest, Hungary

2 HCEMM-SE Cardiometabolic Immunology Research Group, Semmelweis University, Budapest, Hungary

3 MTA-SE Momentum Cardio-Oncology and Cardioimmunology Research Group, Semmelweis University, Budapest, Hungary

4 Ludwig Boltzmann Institute for Cardiovascular Research at the Center for Biomedical Research, Medical University of Vienna, Austria

5 Department of Neurophysiology & Neuropharmacology, Center for Physiology & Pharmacology, Medical University of Vienna, Vienna, Austria

6 Division of Cardiology, Department of Medicine, University of Alberta, Edmonton, Canada

7 Pharmahungary Group, Szeged, Hungary

[*,#] These authors contributed equally to this work.

[†]Address for correspondence

Zoltán V. Varga address: Nagyvarad ter. 4., Budapest, Hungary

e-mail: varga.zoltan@med.semmelweis-univ.hu

Tel: +3612104416

Abbreviations

DMD: Duchenne muscular dystrophy; NF-κB: nuclear factor kappa B; IL-1β: interleukin 1 beta; NLRP3: NLR family, pyrin domain containing 3; AIM2: absent in melanoma 2; NLRC4: NLR family CARD domain-containing protein 4; TNFα: tumor necrosis factor alpha; DAMP, PAMP: danger- or pathogen-associated molecular patterns; IL-18: interleukin-18; ASC: Apoptosis-associated speck-like protein containing a CARD; GSDMD: gasdermin D; MPO: myeloperoxidase; PBS: phosphate buffer saline

SUPPLEMENTARY TABLES

Supplementary Table S1 – List of primary and secondary antibodies used for Western blot and immunohistochemistry

**Method**	**Antibody**	**Dilution**	**Catalogue number**
**Western blot**	AIM2	1:2500	12948S, CST
	NLRP3	1:2500	15101S, CST
	NLRC4	1:2500	12421S, CST
	ASC	1:5000	13833S, CST
	Caspase-1	1:1000	3866S, CST
	IL-1β	1:1000	12703S, CST
	Cleaved-caspase-1	1:1000	4199S, CST
	Cleaved IL-1β	1:1000	83186S, CST

	IL-18	1:1000	HPA003980, Sigma
	Gasdermin D	1:1000	93709S, CST
	GAPDH	1:5000	5174S, CST
	Anti-rabbit IgG (HRP-conjugated)	1:5000	7074S, CST
	Anti-mouse IgG (HRP-conjugated)	1:5000	7076S, CST
**Immunohistochemistry**	CD68	1:500	sc-20060, Santa Cruz
	myeloperoxidase	1:500	AF3667, R&D System

SUPPLEMENTARY FIGURES AND LEGENDS

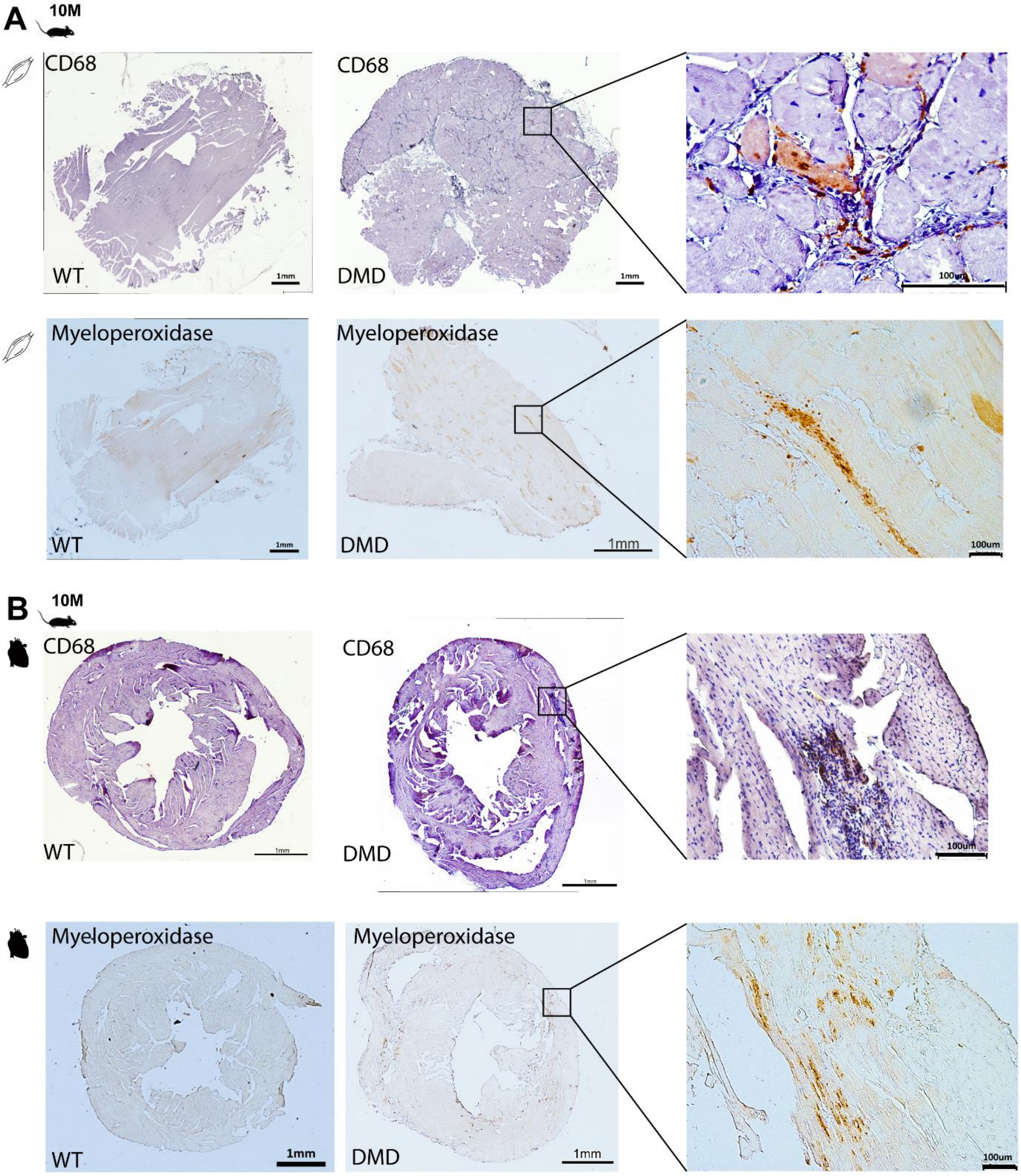

Supplementary Figure S1. Immunohistochemical detection of leukocyte infiltration in skeletal and cardiac muscle samples of mdx mouse at 10 months Representative immunohistochemical images from skeletal muscle (A) and heart tissue (B) samples. Leukocyte subpulations were detected by immunostaining against CD68 or myeloperoxidase

in wildtype and mdx mice. Hematoxyline was used for counterstain in CD68-stained images. Scale bars are indicated on the images (1mm, 100µm).

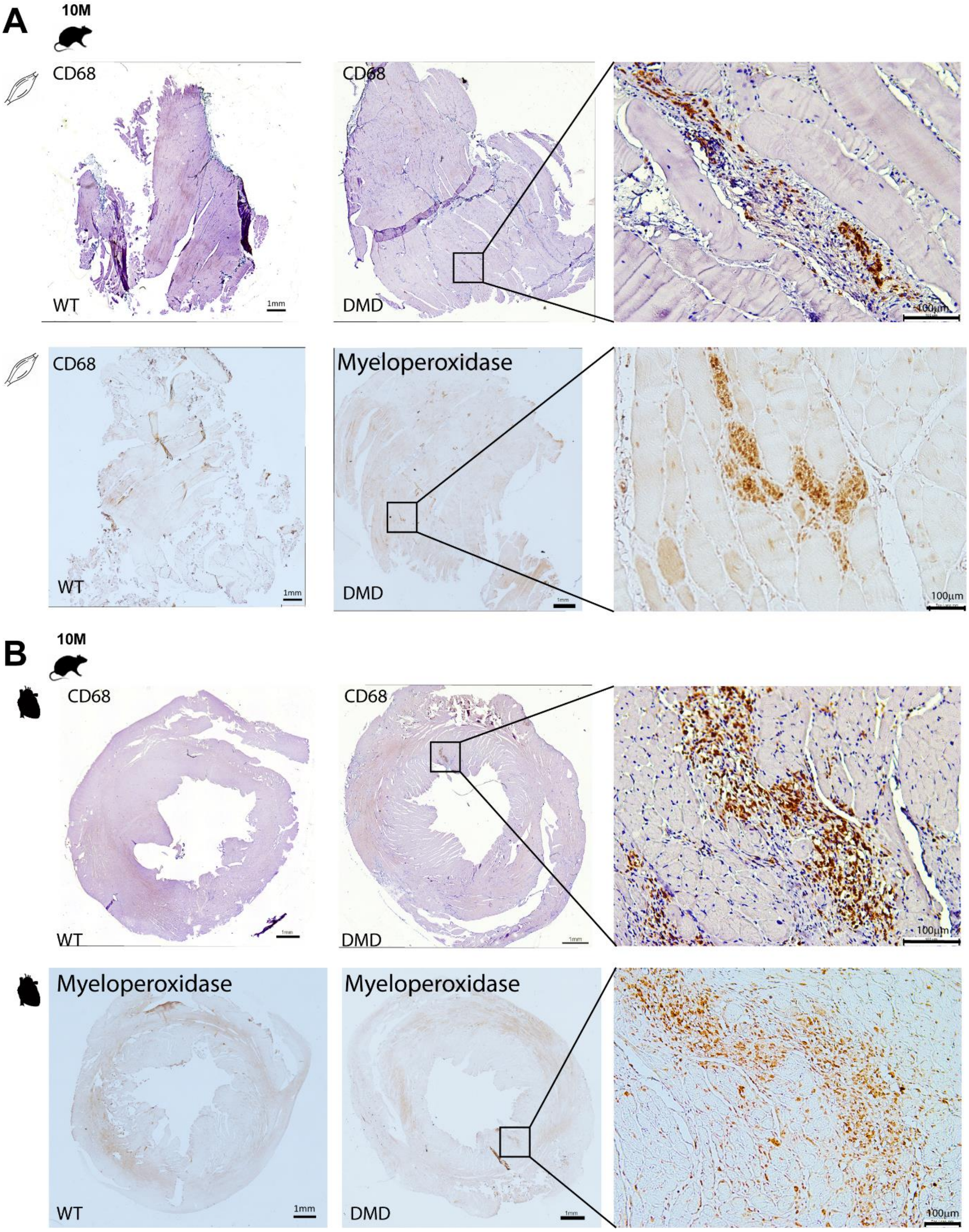

Supplementary Figure S2. Immunohistochemical detection of leukocyte infiltration in skeletal and cardiac muscle samples of DMD^{mdx} rat at 10 months Representative immunohistochemical images from skeletal muscle (A) and heart tissue (B) samples. Leukocyte subpopulations were detected by immunostaining against CD68 or myeloperoxidase in wildtype and DMD rats. Hematoxylin was used for counterstain in CD68-stained images. Scale bars are indicated on the images (1mm, 100µm).
